# Role of *NDP*- and *FZD4*-Related Novel Mutations Identified in Patients with FEVR in Norrin/*β*-Catenin Signaling Pathway

**DOI:** 10.1155/2020/7681926

**Published:** 2020-04-27

**Authors:** Shuai Han, Junhui Sun, Liwei Yang, Ming Qi

**Affiliations:** ^1^Department of Cell Biology and Medical Genetics, School of Medicine, Zhejiang University, Hangzhou 310000, China; ^2^Department of Obstetrics, Zhejiang Provincial People's Hospital, People's Hospital of Hangzhou Medical College, No. 158, Shangtang Road, Hangzhou, Zhejiang Province, China; ^3^Zhejiang California International Nanosystems Institute, Zhejiang University, Hangzhou 310000, China; ^4^DIAN Diagnostics, Hangzhou 310000, China; ^5^Assisted Reproduction Unit, Department of Obstetrics and Gynecology, Department of Laboratory Medicine, Sir Run Run Shaw Hospital, Zhejiang University School of Medicine, Key Laboratory of Reproductive Dysfunction Management of Zhejiang Province, Hangzhou 310000, China; ^6^Department of Pathology and Laboratory Medicine, University of Rochester Medical Center, Rochester, New York 14642, USA

## Abstract

Mutations in *NDP* and *FZD4* have been closely related to a series of retinal diseases including familial exudative vitreoretinopathy (FEVR). Our study was designed to identify novel *NDP* and *FZD4* mutations by whole exome sequencing (WES) in a cohort of patients with a definitive diagnosis of FEVR and explore the underlying molecular mechanism. During 2016, we investigated fifty nonconsanguineous families with affected individuals exhibiting FEVR phenotype and WES identified one recently reported mutation: *NDP* c.127C>A (p.H43N), and five novel mutations: *NDP* c.129_131del (p.44del), *NDP* c.320_353del (p.R107Pfs), *NDP* c.321delG (p.L108Cfs), *ND*P c.377G>T (p.C126F), and *FZD4* c.314T>G (p.M105R) that cosegragated with the abnormal fundus vascular manifestations in six families. All the mutations were perceived to be pathogenic or likely pathogenic according to the standards and guidelines from the American College of Medical Genetics and Genomics (ACMG) and predicted to be deleterious by a series of bioinformatics analyses. We systematically performed functional analyses on the six mutations utilizing the Topflash reporter assay, where all *NDP* and *FZD4* mutants revealed at least 50% loss of wild-type activity. Immunoprecipitation finally demonstrated that the six mutations could degrade the Norrin-Frizzled-4 pair-binding effect to varying degrees. Finally, our study underscores the correlation between the FEVR phenotype and genotype in *NDP* and *FZD4*, extending the mutation spectrum, allowing a reliable assessment of FEVR recurrence and improving genetic counseling. Further, our findings provide essential evidence for the follow-up study of animal models and drug targets by Topflash assays and immunoprecipitation.

## 1. Introduction

Familial exudative vitreoretinopathy (FEVR) is a rare inheritable retinal-vascular disorder characterized by retinal avascularity and first described by Criswick and Schepens in 1969 [1]. Typically, the visual problems of FEVR have been featured by secondary complications such as macular and vascular dragging, tractional retinal detachment, retinal folds, ocular neovascularization, and vitreous hemorrhage [2, 3]. The diverse expressivity of FEVR could be demonstrated as intrafamilial variability and disease asymmetry [4–9].

To our knowledge, numerous investigations have been conducted to date into the pathological mechanism of FEVR, where the canonical Wnt signaling network has been demonstrated to play a pivotal role during retinal organogenesis and angiogenesis [6, 10]. The Norrie disease protein (NDP), Norrin, an unconventional Wnt ligand, coordinated with coreceptors: Frizzled-4 (FZD4), LRP5, and TSPAN12, has been elucidated to induce the Norrin/*β*-catenin canonical signaling pathway, one of the main drivers for retinal angiogenesis in the mammalian eye [6, 11–15]. As Xu et al. described, Norrin with a cysteine-knot motif could bind specifically with the cysteine-rich domain (CRD) of Frizzled-4 and potently activate the classical Wnt signaling pathway [13]. Thus, mutations of *NDP* and *FZD4* in cysteine-knot motif and CRD, respectively, are a good model for elucidating the high affinity of the Norrin-Frizzled-4 pair. Mutations in *NDP* have been associated with X-linked FEVR (MIM#305390), and mutant *FZD4* is linked to an autosomal dominant form of FEVR (MIM#133780). The similar clinical phenotype presented by *NDP* and *FZD4* mutants has also led us to inquire the mechanism of the Norrin-Frizzled-4 pair involved in retinal vasculogenesis.

In our study, we successfully detected one reported mutant and five novel mutants of *NDP* and *FZD4* in fifty Chinese patients with FEVR during 2016 by whole exome sequencing (WES). Then, we showed that FEVR-associated Norrin and Frizzled-4 mutations inhibited the canonical signaling cascade and the possible mechanism: the decrement of Norrin-Frizzled-4 pair affinity, which might implicate the significant phenotypic effect of the patients.

## 2. Materials and Methods

### 2.1. Patient Screening

Fifty patients, who underwent professional ophthalmology examinations and diagnosed as FEVR, were performed WES of peripheral blood genomic DNA during 2016 in Sir Run Run Shaw Hospital, Zhejiang University School of Medicine. Only FEVR patients with novel mutations of *NDP* and *FZD4* were gathered at the time of study initiation. Informed consent was obtained from all participants in the study. This study adhered to the tenets of the Declaration of Helsinki on human subjects and was approved by School of Medicine, Zhejiang University, Hangzhou, China.

### 2.2. Next Generation Sequencing, Data Analysis, and Mutation Validation

Genomic DNA samples were extracted from peripheral blood leukocytes using a QIAGEN QIAamp DNA Blood Mini Kit (Qiagen, Hilden, Germany), subjected to whole-exome capture on Agilent SureSelect Human All Exon V6 Capture (Agilent, California, USA) and performed high-throughput sequencing on Illumina HiSeq 2000 (Illumina, Inc., San Diego, CA, USA). The sequence data were aligned to the human reference genome: University of California, Santa Cruz (UCSC) hg19 (http://genome.ucsc.edu) with Burrows-Wheeler aligner version 0.7.10: BWA-MEM (http://biobwa.sourceforge.net/). Further, we calibrated variants using the Genomic Analysis Toolkit (https://software.broadinstitute.org/gatk/) and conducted functional annotation by Annovar (http://www.openbioinformatics.org/annovar/) and SnpEff (http://www.snpeff.sourceforge.net). The benign variants were filtered with minor allele frequency (MAF) > 1% in the 1000 Genomes data set (1000G) (https://www.internationalgenome.org/1000-genomes-browsers/), the Single Nucleotide Polymorphism (dbSNP) (http://www.ncbi.nlm.nih.gov/SNP.), Genome Aggregation Database (gnomAD) (http://gnomad.broadinstitute.org/), Exome Aggregation Consortium (ExAC) (http://exac.broadinstitute.org/), and our internal database. Human Gene Mutation Database (HGMD) (http://www.hgmd.cf.ac.uk/ac/index.php), ClinVar (http://www.ncbi.nlm.nih.gov/clinvar/), Online Mendelian Inheritance in Man (OMIM) (https://OMIM.org), and Leiden Open Variation Database (LOVD) (http://lovd.nl) were used to annotate the existence of mutation reports. The silico analyses of the missense variants were predicted by PolyPhen-2 (http://genetics.bwh.harvard.edu/pph2/), Sorting Intolerant From Tolerant (http://sift-dna.org), and Mutation Taster (http://www.mutationtaster.org/). We performed Sanger sequencing to validate the suspicious variants and confirm the segregation of the identified variants in the affected and unaffected family members. PCR primers were designed as follows: *NDP*-127/129-131-F: TTCCTTGAACGGGACTGGAT; *NDP*-127/129-131-R: AGCCTCATTCTCCCACAAG; *NDP*-320-353/321/377-F: GCAACGAGTGTGAGGGTCTT; *NDP*-320-353/321/377-R: CCCAAACAGCATTGAGAGCC; *FZD4*-314-F: AACTCAGCTTTGTGGGAGCA; *FZD4*-314-R: AATATGATGGGGCGCTCAGG.

### 2.3. 3D Modeling of *NDP* and *FZD4* Missense Changes

We adopted University of California, San Francisco (UCSF) chimera software to mutate the residue identified in our study according to three-dimensional structure of Norrin and Frizzled-4 (PDB: 5pqc/5pqe).

### 2.4. Construction of Expression Plasmids

Wild-type *NDP* cDNA was purchased from Kelei Biological Technology Co., Ltd. (Shanghai, China) and amplified using forward 5′-CCGctcgagCGGATGAGAAAACATGTACTAGCTG-3′ and reverse primer 5′-CGggatccCGGGAATTGCATTCCTCGCAGTGA-3′. Then, we subcloned wild-type *NDP* cDNA into pEGFP-N1 vector (BD Biosciences) using XhoI and BamHI sites. All *NDP* mutants were introduced into the wild-type *NDP* cDNA by primer-mediated PCR mutagenesis. It should be noted that for deletion frameshift mutations (*NDP* c.321del/320_353del), in order not to affect the subsequent expression of EGFP, the reverse primer was designed as follows: 5′-CGggatccGGAATTGCATTCCTCGCAGTGA-3′.

The recombinant plasmids containing NDP-EGFP fusion constructs were verified by direct DNA sequencing and then amplified and purified for transfection (Qiagen Inc., Valencia, CA). Construction of wild-type and mutant pCMV-3xFlag-FZD4 recombinant plasmids was described as above using HindIII and BamHI sites. The corresponding primers were as follows: forward 5′-aagcttATGGCCTGGCGGGGCGCA-3′ and reverse primer 5′-CggattcCGTACCACAGTCTCACTG-3′.

The primers for site-directed mutagenesis were *NDP*127-mutant-f: TGCATGAGGCACaACTATGTGGATT; *NDP* 127-mutant-r: ACGTACTCCGTGtTGATACACCTAA; *NDP* 129_131-mutant-f: CATGAGGCAC(cta)CATGTGGATTCTAT; *NDP* 129_131-mutant-r: GTACTCCGTGGT(gat)ACACCTAAG ATA; *NDP* 320_353-mutant-f: GCACTGC(…)CACCTACCGGTACATCCTCT; *NDP* 320_131-mutant-r: CGGTAGGTG(…)GCAGTGCCTTCAGCTTGG; *NDP* 321-mutant-f: AGGCACTGCG(g) CTGCGATGCT; *NDP* 321-mutant-r: TCCGTGACGC(c) GACGCTACGA; *NDP* 377-mutant-f: CATCCTCTCCTtTCA CTGCGAG; *NDP* 377-mutant-r: GTAGGAGAGGaAGT GACGCTC; *FZD4* 314-mutant-f: TTTATGTGCCAAgGTGCACAGAGAA; *FZD4* 314-mutant-r: TTCTCTGTGCACcTTGGCACATAAA.

### 2.5. Topflash Report Assay

To generate HEK 293 cell line harboring SuperTopFlash (STF) reporter gene, 125 ng of the STF construct (Kelei Biological Technology Co., Ltd., Shanghai, China) and 125 ng of pSV2neo (Kelei Biological Technology Co., Ltd., Shanghai, China) providing a neomarker for dominant selections screening out by 600 *μ*g/ml of G418 (Invitrogen Corporation, Carlsbad, California, USA) were stably transfected into HEK 293 cells in a 48-well plate using PolyJet (Invitrogen Corporation, Carlsbad, California, USA) [16]. The HEK 293 cell lines harboring STF reporter gene were cotransfected with 200 ng of Norrin (wild type or mutant), 200 ng of FZD4 (wild type or mutant), 200 ng of LRP5, and 100 ng of pSV-*β*-gal (Kelei Biological Technology Co., Ltd., Shanghai, China) in a 48-well plate using PolyJet (Invitrogen Corporation, Carlsbad, California, USA). Forty-eight hours after transfection, we washed the cells twice with 1x PBS and subjected to luciferase assay using Dual Luciferase assay reagent (Promega, USA).

### 2.6. Western Blotting

Western blotting was performed as described previously using *β*-catenin monoclonal antibody (1: 1000, Beyotime AF0069) [17].

### 2.7. Generation of Norrin-Conditioned Medium

HEK 293T cells were seeded at a density of 0.8 × 10^6^ in six 10 cm^2^ plates and transfected with mutant or wild-type *NDP*-EGFP fusion constructs. Sixteen hours later, the medium was removed and replaced with fresh medium. After thirty-six hours, the medium was neutralized by adding sterile 1 M HEPES pH 8.0 and conditioned for additional one day. The medium was concentrated in Amicon Ultra 3K Millipore.

### 2.8. Native Polyacrylamide Gel Electrophoresis

Plasma Membrane Protein Isolation and Cell Fractionation Kit (Invent Biotechnologies, Inc., Eden Prairie, USA) was used to isolated membrane proteins. A NativePAGE Preparation Kit (Sangon Biotech, Shanghai, China) was adopted to make up a standard 8% polyacrylamide gel, which was loaded (without SDS and reducing reagent) with 8 *μ*l of corresponding conditioned medium for each construct tested. Electrophoresis was carried out using as described by Qin et al. [18].

### 2.9. Immunoprecipitation

We transfected HEK 293T cells from a 60 mm^2^ plate with wild-type FZD4-3xFlag constructs or mutants, which were subsequently incubated with 3 ml of the mutant or wild-type Norrin conditional medium on ice for one hour. Subsequent immunoprecipitation was performed using GFP monoclonal antibody (1 : 1000, Beyotime AF0159) and Flag monoclonal antibody (1 : 1000, Beyotime AF5051) as described by Xu et al. [13].

## 3. Results

### 3.1. Novel Mutations Identified in *NDP* and *FZD4* and Bioinformatics Prediction

**Figure 1:**
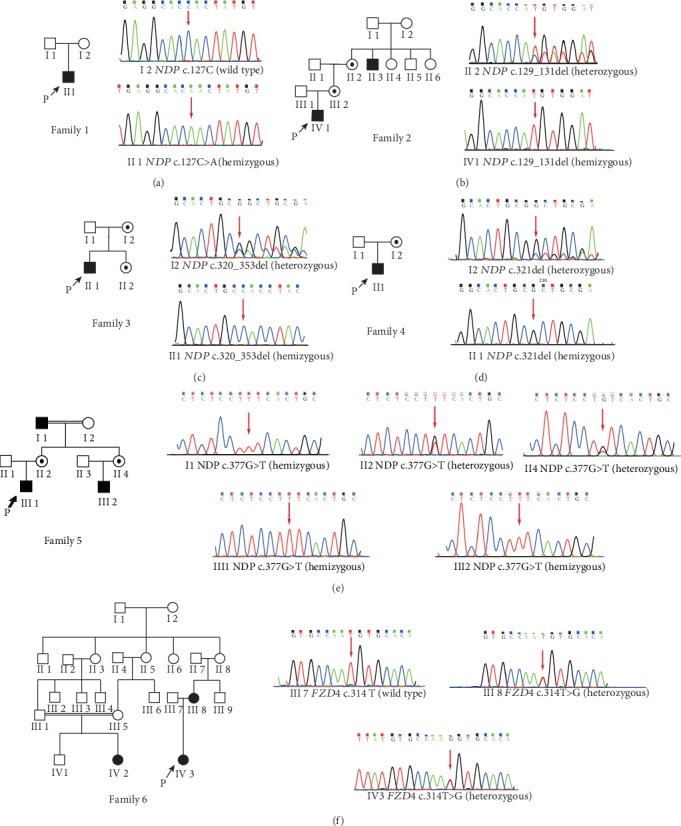
Pedigrees and DNA sequences. (a) Pedigree of family 1. The sequencing of the proband and his mother. (b) Pedigree of family 2. The sequencing of the proband and his mother. (c) Pedigree of family 3. The sequencing of the proband and his mother. (d) Pedigree of family 4. The sequencing of the proband and his mother. (e) Pedigree of family 5. The sequencing of the proband and his mother, aunt, grandfather, and cousin. (f) Pedigree of family 6. The sequencing of the proband and his father and mother. The arrow showed the proband. The red arrow indicated the mutation.

**Figure 2:**
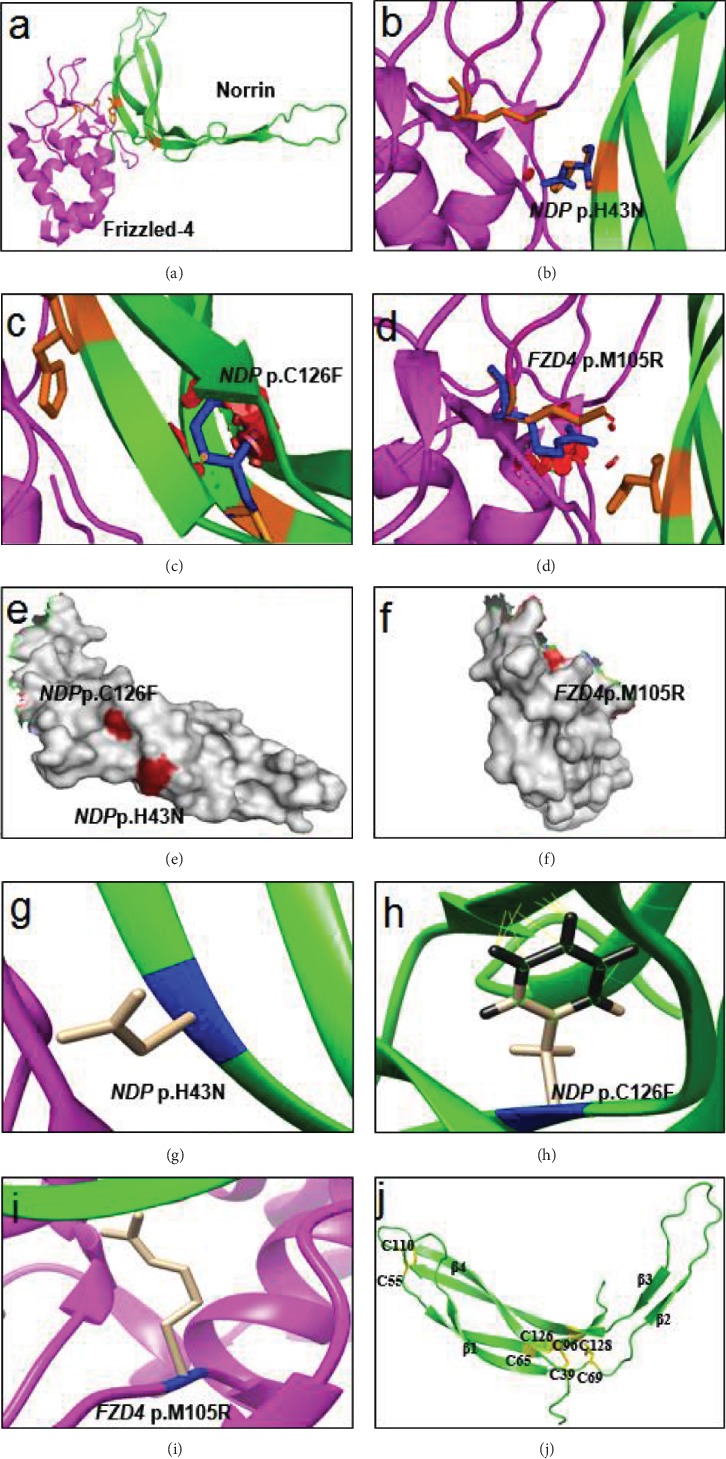
3D modeling of *NDP* and *FZD4* missense changes. (a) Ribbon model of Norrin and Frizzled-4. The wild-type residues of *NDP* and *FZD4* missense mutations were depicted in orange. (b–d) Closeup of *NDP* p.H43N, *ND*P p.C126F, and *FZD4* p.M105R. The most stable confirmation was represented in blue. (e, f) *NDP* and *FZD4* missense mutations were located at the protein interface. (g) After minimizing the protein energy, *NDP* p.H43N could not produce clash/contact bonds. (h) After minimizing the protein energy, *ND*P p.C126F produced clash/contact bonds, which were shown in yellow. (i) After minimizing the protein energy, *FZD4* p.M105R could not produce clash/contact bonds. (j) The cysteine side chains that participated in forming disulfide bonds and creating the critical knot motif were shown in yellow.

Six novel mutations of *NDP* and *FZD4* were identified by WES from six unrelated FEVR probands among fifty nonconsanguineous patients, validated by Sanger sequencing during 2016 (Figure 1). However, in the performance of the following functional experiments, *NDP* c.127C>A (p.H43N) had been reported by Rao et al. in May 2017 [19]. All mutations were proved to be cosegregated with the abnormal phenotype of FEVR probands and perceived as pathogenic or likely pathogenic in accordance with the American College of Medical Genetics and Genomics (ACMG) guidelines, where *NDP* c.127C>A (p.H43N), *NDP* c.129_131del (p.44del), *NDP* c.320_353del (p.R107Pfs), *NDP* c.321delG (p.L108Cfs), and *FZD4* c.314T>G (p.M105R) were graded as pathogenic and *ND*P c.377G>T (p.C126F) was graded as likely pathogenic (Table 1) [20]. Protein sequence alignment of human Norrin/Frizzled-4 with homologues from human and other species indicated that *NDP* p.H43N, *ND*P p.C126F, and *FZD4* p.M105R were nonconservative substitutions (Figure S1). *NDP* p.H43N, *ND*P p.C126F, and *FZD4* p.M105R resided in the Norrin-Frizzled-4 binding area and at the protein interface (Figures 2(a)–2(f)). Notably, *ND*P p.C126F was involved in the second disulfide bond of cysteine knot structure, which were one of the highly conserved trio of disulfide bonds, so any other amino acid at this position would severely hinder the formation of the knot (Figures 2(h) and 2(j)) [21].

### 3.2. Defective Norrin/*β*-Catenin Signaling

**Figure 3:**
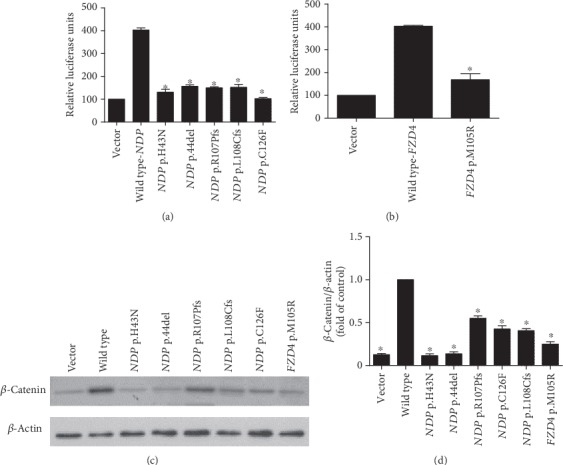
Topflash reporter assay and Western blotting. (a, b) Failure of *NDP* and *FZD4* mutants in the activation of Norrin/*β*-catenin signaling (72.5% reduction for *NDP* p.H43N, 70% reduction for *NDP* p.44del, 71.2% reduction for *NDP* p.R107Pfs, 70.5% reduction for *NDP* p.L108Cfs, 73% reduction for *ND*P p.C126F, and 65% reduction for *FZD4* p.M105R). The experiment was carried out in triplicate at the same time and repeated three times. (c, d) Western blotting showed the expression level of *β*-catenin was decreased, similar to the Topflash reporter assay. Asterisks indicated significant differences from the positive control by pairwise Student's test (*P* < 0.05).

To verify the effect of the six mutations on the Norrin/*β*-catenin signaling, a Wnt-responsive Topflash reporter assay was performed to elucidate the activation of canonical Norrin/*β*-catenin signaling. As a result, all *NDP* and *FZD4* mutants revealed at least 50% loss of wild-type activity (72.5% for *NDP* p.H43N, 70% for *NDP* p.44del, 71.2% for *NDP* p.R107Pfs, 70.5% for *NDP* p.L108Cfs, 73% for *ND*P p.C126F, and 65% for *FZD4* p.M105R) (Figures 3(a) and 3(b)). The expression of *β*-catenin was also detected after the transfection of the corresponding wild-type and mutant plasmids to HEK 293T cells, where the obvious reduction was also consistent with the FEVR presentations (Figures 3(c) and 3(d)). The attenuation of the activity of the Norrin/*β*-catenin signaling strongly proved that the six mutations were pathogenic.

### 3.3. Defective Norrin and Frizzled-4 Binding

**Figure 4:**
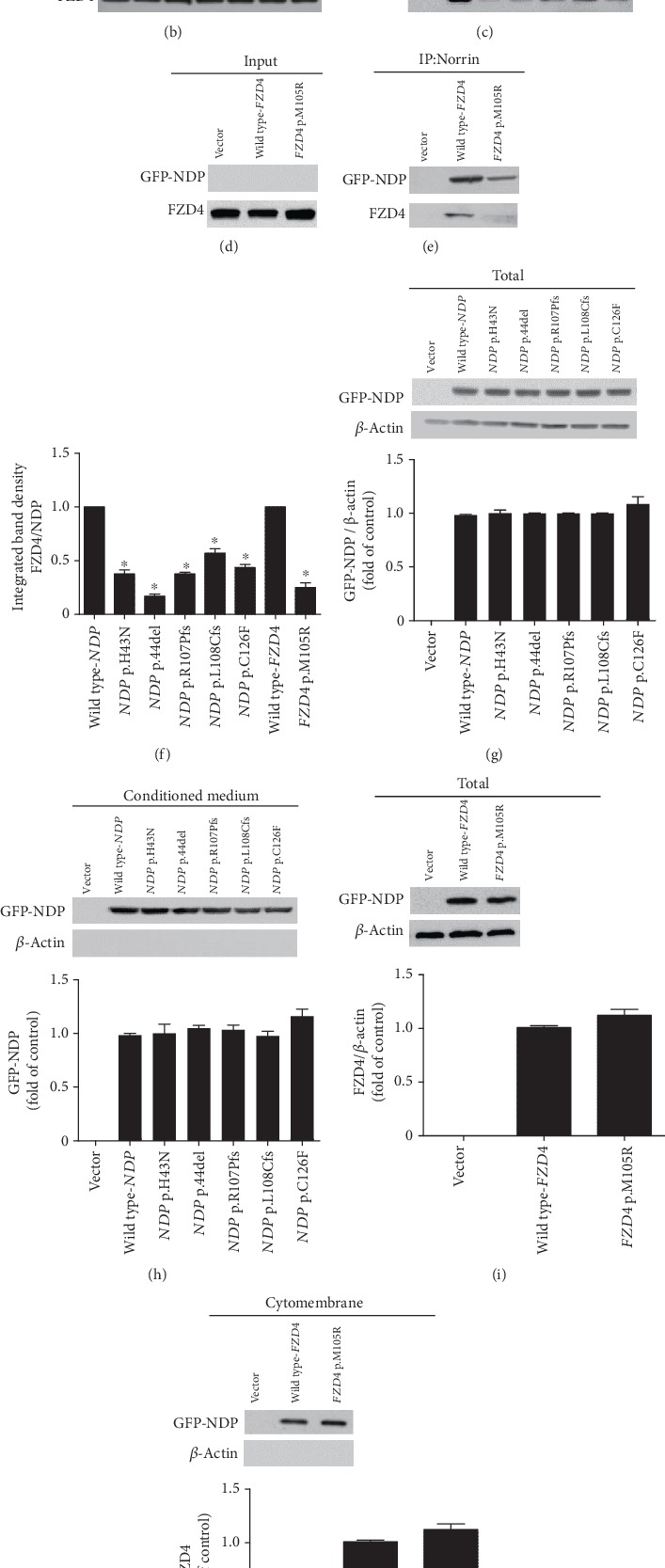
FEVR-linked *NDP* and *FZD4* mutations impaired the binding of Norrin and Frizzled-4. (a) The schematic diagram showed that all the six mutations were located in the CTCK or CRD domain, respectively. (b, c) Transfected cells expressing Frizzled-4 were incubated with wild-type and mutant Norrin-conditioned medium before the Norrin-Frizzled-4 binding complex was immunoprecipitated. Mutant Norrin coprecipitated with Frizzled-4 to varying reduced degrees. It was noted that Norrin concentrations in the diluted lysate were below the detection limit of standard detection systems. (d, e) Transfected cells expressing wild-type and mutant Frizzled-4 were incubated with Norrin-conditioned medium before the Norrin-Frizzled-4 binding complex was immunoprecipitated. Norrin coprecipitated with mutant Frizzled-4 to a distinct reduced degree. It was noted that Norrin concentrations in the diluted lysate were below the detection limit of standard detection systems. (f) Integrated band density quantification of Frizzled-4 coprecipitated with Norrin. (g) Mutant Norrin was expressed at the same level as wild-type Norrin. (h) The secretion of extracellular mutant Norrin was expressed at the same level as wild-type Norrin. (i) Mutant Frizzled-4 was expressed at the same level as wild-type Frizzled-4. (j) Mutant Frizzled-4 was efficiently located to the plasma membrane the same as wild-type Frizzled-4.

Norrin and Frizzled-4 are a high-affinity ligand-receptor pair, which could be used to isolate each other from the plasma membrane [13]. Norrin harbors the cysteine knot domain, which is cysteine-rich and involved in extracellular signaling transduction. Five *NDP*-related mutations were located in the domain, where most reported pathogenic mutations also resided (Figure 4(a)). Moreover, the specificity of Norrin-Frizzled-4 binding has been assessed systematically, and the CRD of Frizzled-4 is the only domain which Norrin binds to. *FZD4* p.M105R was located in the CRD functional domain (Figure 4(a)). To investigate the mechanism of defective Norrin/*β*-catenin signaling and further substantiate the interaction of Norrin and Frizzled-4 CRD binding, immunoprecipitation was performed. All *NDP* mutants bound Frizzled-4 at a severalfold lower level as determined by immunoblotting with anti-GFP and anti-Flag antibodies (Figures 4(b), 4(c), and 4(f)). Moreover, little mutant Frizzled-4 could precipitate with Norrin (Figures 4(d)–4(f)). Obviously, the binding effect was severely reduced, which correlated well with the extent of functional defects observed in the Topflash assays. Native polyacrylamide gel electrophoresis confirmed that the same extracellular secretion amount of mutant and wild-type Norrin was incubated with Frizzled-4 and revealed a normal Frizzled-4 yield of the HEK 293T cytomembrane between the wild-type and mutant Frizzled-4, indicating that the localization of mutant Frizzled-4 at plasma membrane was not affected (Figures 4(g)–4(j)).

## 4. Discussion

In our study, we identified one reported mutation: *NDP* c.127C>A (p.H43N), and five novel FEVR-linked mutations: *NDP* c.129_131del (p.44del), *NDP* c.320_353del (p.R107Pfs), *NDP* c.321delG (p.L108Cfs), *ND*P c.377G>T (p.C126F), and *FZD4* c.314T>G (p.M105R) by WES among fifty nonconsanguineous FEVR families during 2016 and excluded the presence of these mutations in 200 ethnically matched control individuals. It is critical to unravel the defective mechanism of FEVR-linked mutations identified in FEVR patients with genetic heterogeneity. Therefore, to assess if the missense mutations were important for Norrin/*β*-catenin signaling, we first utilized bioinformatics tools to predict to be deleterious preliminarily. Finally, by Topflash assays and immunoprecipitation, we strongly proved that the Norrin and Frizzled-4 binding sensitivity of the six mutations was severely compromised and further impaired the Norrin/*β*-catenin signaling, confirming the link between the mutants in *NDP* and *FZD4* and abnormal retinal presentation.

Previous studies have demonstrated the variable impaired Norrin signal induced by different mutations observed regardless of the disease phenotype [13, 18, 22, 23]. Thus, the finding that the *NDP* and *FZD4* mutations match the significant phenotypic effects could sufficiently evidence the presumed haploinsufficiency. It has been elucidated that *NDP* p.H43R and *NDP* H43Q are associated with Norrie (MIM#310600), an inherited retinal disorder of highly overlapping ocular manifestations with FEVR [24, 25]. Notably, in our study, *NDP* p.H43N was found to be related to FEVR, which was a novel mutation when we conducted the research in 2016, was later reported by Rao et al. in May 2017, and was less characterized in the literature [19]. Thus, the reasons for different diseases caused by different substitutions of the amino acid at the same position also need to be explored. As our knowledge, Norrin function depends on three pairs of cysteines, forming the highly conserved disulfide bonds: Cys^65^-Cys^126^, Cys^69^-Cys^128^, and Cys^39^-Cys^96^, for which any substitution could possibly compromise signaling and Norrin-Frizzled-4 CRD binding, consistent with the significant decrement of signaling and binding activity induced by *ND*P p.C126F [21]. As for *NDP* p.R107Pfs and *NDP* p.L108Cfs, the subsequent amino acids' substitutions might imply the conformational changes of the *β* sheet of Norrin and dimerization, which still need further investigation to clarify. However, there are some discrepancies between the binding defects of *FZD4* M105V to Norrin, located at the same position as *FZD4* p.M105R and reported by Xu et al. and Qin et al., where the former describe a normal production at plasma membrane and the latter detect obvious binding defects [13, 18].

So far, there have been at least nine genes attributed to the progression of FEVR including *NDP* [26], *FZD4* [27], *LRP5* [28], *TSPAN12* [29], *ZNF408* [30], *KIF11* [31], *RCBTB*1 [32], *CTNNB1* [33], and *JAG1* [34]. Our previous results also provided insight into the relationship between a novel splicing mutation (c.734+1G>A) in *CTNNB1* and a 27-year-old Chinese pregnant woman with a severe intellectual disability and FEVR [35]. Human FEVR displays variable ocular defects and serves as an excellent model to explore Wnt signaling, especially causing mutations in *NDP*, *FZD4*, *LRP5*, and *TSPAN12*. To date, the Norrin-Frizzled-4 signaling system has been clarified to play a central role in retinal vascular development and blood-brain barrier plasticity [13, 36]. Luhmann et al. showed that *NDP*-knockout mice could occur in the regression of the hyaloid vascular alongside the delay of the superficial retinal vasculature and the failure of the deep retinal vasculature [37]. Similarly, *FZD4*-knockout mice have been shown defective in vasculature in the retina and inner ear [13]. Xia et al. verify that *LRP5*-knockout mice develop capillary network incompletely and capillaries lacking lumen structure [38]. Afterwards, the formation of microaneurysms, aberrant fenestration, and delayed hyaloid vessel regression could be observed in the *TSPAN12*-knockout mice [12]. Subsequently, further research on the role of Norrin/*β*-catenin in the retinal vascular induced by the mutations detected in our study would be carried out using animal models.

## 5. Conclusions

Pathogenic mutations in *NDP* and *FZD4* could lead to a series of retina-related diseases such as FEVR, Norrie, and retinopathy of prematurity (MIM#133780), which could be distinguished according to their unique characteristics. Furthermore, our present work represents a step in representing points along the FEVR phenotypic spectrum. Taken together, these findings would allow a reliable assessment of FEVR recurrence and improve genetic counseling. We believe that our findings would provide essential evidence for the follow-up study of animal models and drug targets, as well as for understanding the underlying molecular mechanism of FEVR [39].

## Figures and Tables

**Table 1:** Identified mutations of *NDP* and *FZD4* in six families with FEVR.

Family	Gene	Type	Mutation	1000G	ExAC	gnomAD	Cosegregation	ACMG
Family 1	*NDP*	Missense	c.127C>A, p.H43N	Novel	Novel	Novel	De novo	Pathogenic
Family 2	*NDP*	Nonframeshift	c.129_131del, p.44del	Novel	Novel	Novel	Maternal	Pathogenic
Family 3	*NDP*	Frameshift	c.320_353del, p.R107Pfs	Novel	Novel	Novel	Maternal	Pathogenic
Family 4	*NDP*	Frameshift	c.321delG, p.L108Cfs	Novel	Novel	Novel	Maternal	Pathogenic
Family 5	*NDP*	Missense	c.377G>T, p.C126F	Novel	Novel	Novel	Maternal	Likely pathogenic
Family 6	*FZD4*	Missense	c.314T>G, p.M105R	Novel	Novel	Novel	Maternal	Pathogenic

## Data Availability

The data used to support the findings of this study are included within the article.

## Supplementary Materials

Supplementary Materials Figure S1: Norrin and Frizzled-4 sequence alignment of missense mutations.
