# Supplementary material for: Role of NDP- and FZD4-Related Novel Mutations Identified in Patients with FEVR in Norrin/β-Catenin Signaling Pathway
Source: Biomed Res Int. 2020 Apr 27;2020:7681926. doi: 10.1155/2020/7681926 (PMC7201721; doi:10.1155/2020/7681926)
Supplement: Supplementary Materials — Figure S1: Norrin and Frizzled-4 sequence alignment of missense mutations. [file 7681926.f1.docx]

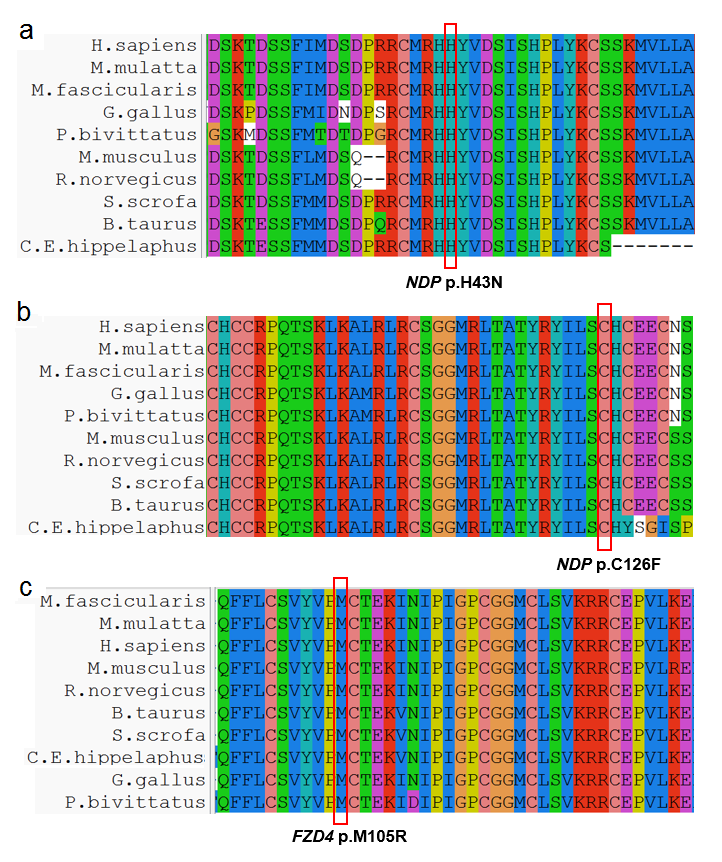


Figure.S1 Norrin and Frizzled-4 sequence alignment of missense mutations.

a, b, c: An amino acid sequence alignment of Homo Sapiens, Mus muscμlus, Cervus elaphus hippelaphus, Rattus norvegicus, Gallus gallus, Bos taurus, Macaca fascicμlaris, Sus scrofa, Macaca mμlatta, Python bivittatus indicated that that *NDP* p.H43N, *ND*P p.C126F and *FZD4* p.M105R were non-conservative substitutions.
